# Using a co‐production prioritization exercise involving South Asian children, young people and their families to identify health priorities requiring further research and public awareness

**DOI:** 10.1111/hex.12524

**Published:** 2016-12-08

**Authors:** Logan Manikam, Rakhee Shah, Kate Reed, Gupreet Santini, Monica Lakhanpaul

**Affiliations:** ^1^ Population, Policy and Practice UCL Great Ormond Street Institute of Child Health London UK; ^2^ South Asian Health Foundation Birmingham UK; ^3^ Royal London Hospital London UK; ^4^ KCL Faculty of Life Sciences & Medicine London UK; ^5^ Action on Hearing Loss Leicester UK

**Keywords:** co‐production, health priorities, marginalized communities, prioritization exercise, South Asians

## Abstract

**Objectives:**

To facilitate South Asian (SA) families and health‐care professionals (HCPs) participation in a prioritization exercise to co‐produce child health research and public awareness agendas.

**Design:**

A three‐stage process was adopted involving the following: (i) systematic literature review, (ii) HCP scoping survey and (iii) focus groups of SA adolescents and families. A Punjabi‐ and Urdu‐speaking community facilitator moderated focus groups. A British Sign Language interpreter assisted in the hard of hearing group. Concordant and discordant themes between HCPs and SAs were identified.

**Setting:**

National survey of HCPs. Leicestershire for SA families.

**Participants:**

A total of 27 HCPs and 35 SAs. SAs varied by descent, age (16‐74), UK stay length (3‐57 years) religion and disability.

**Results:**

Ranked by submission frequency in the survey, HCPs prioritized (i) public awareness on obesity, mental health, health‐care access, vitamin D and routine health checks and (ii) research on nutrition, diabetes, health education and parenting methods.

**Discussion:**

South Asians prioritized research into the effectiveness of alternative medicines, a theme not identified by HCPs. Both HCPs and SAs prioritized increased research or public awareness on mental health illness, blood and organ donation, obesity and diet. Whilst HCPs identified diabetes, vitamin D and rickets together with parenting methods were important priorities requiring increased public awareness, and these views were not shared by SAs.

**Conclusions:**

Minority groups are not always included in priority setting exercises due to concerns about language and perceived difficulty with accessing communities. Through this co‐production exercise, we showed that it is possible and essential.

## 1 INTRODUCTION

Marginalized communities are often excluded from prioritization exercises due to concerns about language barriers or accessing communities.^1^, ^2^, ^3^ It is increasingly being recognized that health interventions, public awareness campaigns and research that translates to health improvements should be co‐produced with patients and families.

At a time of limited resources, prioritization of research topics is a necessary part of the research process and subsequent health‐care commissioning.^4^ By encouraging co‐learning and mutual ownership of the products of the research collaboration, it is argued that this will improve research quality by greater participation rates, increase external validity and decrease loss of follow‐up.^5^, ^6^

Co‐production of research agendas may lead to more effective and efficient interventions in addition to better health outcomes. Involving the public as research partners will begin to see the long‐term gains associated with research that will facilitate quicker translation into routine clinical practice.^3^, ^4^ This also ensures that both financial and non‐financial resources are not wasted on research that is either not useful or relevant to a community.^7^, ^8^, ^9^

With these proclaimed benefits, public involvement in decision making is increasingly common with particular emphasis on patient perspectives and collaborative processes.^8^, ^10^, ^11^, ^12^, ^13^ In several systematic reviews, it has been noted that research, involving patients as active contributors, has grown from a paltry six publications to 27 and 150 in 2010 and 2012, respectively.^8^, ^14^ Expansion of public involvement in critiquing systematic review methods and outcome measures selected has also been noted.^6^

Increasingly, a shift towards initiatives that encourage partnerships between health professionals and the public to jointly identify and prioritize research by facilitated debate and formal decision‐making methods has been seen.^15^ Key examples of this include the National Institute for Health Research Health Technology Appraisal Programme that began incorporating the public as panel members or external experts since 1997.^16^ As a consequence, the programme's commissioned research is now positively influenced with explicit patient and carer perspectives, more relevant research focus and outcomes, and it provides plain English background text.^17^, ^18^

### 1.1 Co‐production with ethnic minorities

Increasingly policy‐makers, researchers and HCPs are advocated to examine and adopt research and health priorities to meet the specific needs of ethnic minority populations.^19^ This is iterated in the public health strategy “Healthy Lives, Healthy People” which recommends an innovative and responsive approach that is owned by communities and shaped by their needs to bring about real change.^20^

The benefits of joint prioritization can be viewed as being particularly critical in ethnic minority populations, where research formulated without the input, and involvement of these communities can undermine the research and its success from the outset.^21^ Improving outcomes for these populations therefore requires input across the whole transitional pathway from research to service delivery and public awareness.

### 1.2 SAs in the United Kingdom

SAs are a heterogeneous group of individuals of Indian, Pakistani, Bangladeshi and Sri Lankan origin, with differing religion, language and culture. They live across the United Kingdom (UK), with clustering in certain areas reflecting migration patterns.^22^ In the most recent UK Office of National Statistics Census (2011), it was noted that SAs made up 5.3% of the UK population, with SA countries continuing to rank highly as the most common non‐UK countries of birth.^23^ SAs are marginalized in the UK because of their access to and use of health care.^24^ Barriers include inadequate information, unfamiliarity with health‐care systems, language barriers, insufficient support in interpreting and translating with limited fluency in English and confusion around entitlement to some types of services.^24^, ^25^ Other factors include cultural reasons such as the use of complementary therapy,^26^, ^27^, ^28^ stigma for using mental health services,^29^ and lower socio‐economic status.^25^ However, despite SAs making up 5.3% of the UK population, it is recognized that engaging minority communities in research is still limited.^1^, ^30^ It is therefore important to give marginalized communities a voice and hear what matters to them to try and minimize health inequalities.

It is well‐known that both SA adults and children have different health needs when compared to their White British counterparts. Examples include differences in rates of acute asthma admissions, psychiatric morbidity, type 2 diabetes and cardiovascular disease.^21^, ^31^, ^32^

A growing evidence base suggests that these differences are attributable to ethnic variations in disease severity, differences in health‐seeking behaviour and/or health service accessibility.^19^, ^21^, ^31^ This however is not an exhaustive list; increased prevalence of genetic conditions due to social influences, vitamin D deficiency due to differing nutritional intake and lack of organ transplantations due to cultural issues are notably other differences.^33^, ^34^, ^35^ Involving these marginalized communities, using methods adapted from other studies such as Social Action Research or Participation Action Research, will therefore identify factors in lifestyle, for example, that lead to differing health outcomes.^2^, ^3^, ^35^, ^36^

In the light of the limited understanding of the research agendas of SAs, the South Asian Health Foundation (SAHF) initiated the first study to involve both SAs and HCPs in identifying priorities for investment in research and public awareness priorities and to identify outcome indicators important to SAs that researchers could use to measure improvements in health. This study presents the key methodology and findings from this work.

## 2 METHODS

Informed by a health psychologist and experts in prioritization exercises, our exercise consisted of three phases: (i) a systematic literature review of prioritization exercise methodology to inform our exercise and published SA health/research/public awareness priorities, (ii) scoping survey of HCPs to build on existing and identify any further SA unpublished public health and research priorities and (iii) focus groups of SAs to discuss and rank these priorities.

Two lists of health topics requiring increased awareness and research were produced, one for topics prioritized by HCPs and one for topics prioritized by SAs. Similarities and differences between both lists were identified and presented.

### 2.1 Systematic literature review

Wide methodological variability and the spectrum of stakeholder engagement can significantly affect both the credibility of the prioritization process and subsequent translation into research commissioning.^4^

A systematic literature review was therefore undertaken to identify the following: (i) prioritization exercise methodologies, (ii) collaborative methods used with children and/or their families and with SAs, (iii) health topics and/or outcomes about SAs significant to health‐care professionals (HCPs) or SAs and (iv) if and when health differences in SA subpopulations matter in priority settings.

This together with input from a health psychologist informed the subsequent development of a tailored culturally appropriate method to undertake a prioritization exercise involving SA adolescents, their families and HCPs.

#### 2.1.1 Search strategy

The search strategy was derived in collaboration with a London School of Hygiene and Tropical Medicine Information scientist. The strategy included terms for “SA” and “children” and specified all major subgroups with either free text or Medical Subject Heading (MeSH) terms. For example, the search terms used for prioritization methodologies were needs, outcome or process assessment, health or research priorities. In contrast, topic scoping search terms included patient or consumer participation, patient or consumer advocacy, health or research priorities and outcomes. These were combined with population terms that included South Asian, India, Pakistan, Bangladesh, Sri Lanka, ISC, child, young person or adolescents.

#### 2.1.2 Information sources

A single author screened titles and abstracts for relevance. Both qualitative and quantitative studies were included. The databases searched were MEDLINE, EMBASE, COCHRANE LIBRARY and OpenSIGLE. Databases were searched via the OvidSP for evidence between 1990 and 2014. Internet search engines such as Google Scholar were utilized. Additionally, the following specialist organization websites were also searched for grey literature: Royal College of Paediatrics and Child Health (RCPCH), National Health Service (NHS) Evidence, James Lind Alliance (JLA), National Institute for Health Research (NIHR), INVOLVE and SAHF. Studies were included if they met any of the four inclusion criteria (i)‐(iv) specified above and excluded if they were either non‐extractable or not published in English.

### 2.2 Scoping survey of HCPs

To provide a more exhaustive picture of public health and research priorities than what was available from published sources, a scoping survey of HCPs with an interest in SA health was undertaken. The scoping survey was limited to HCPs only and not applied to the SA community because of the anticipated difficulties in accessing local communities electronically. The scoping survey was therefore developed to identify the health topics important to HCPs requiring increased research or public awareness.

Survey development was informed by the literature review and consultation with an independent health psychologist. It was piloted in a small group of professionals to assess readability and consistency of responses.

In each, respondents were asked to list five topics related to SA children that they felt were (i) under‐researched and should be priority areas for research, (ii) needed to be promoted to raise public awareness and (iii) relevant outcome indicators that should be measured to demonstrate success of any health intervention.

The James Lind Alliance methodology of priority setting is an initiative that brings patients, carers and clinicians together in priority setting partnerships to prioritize uncertainties in treatments.^37^ In line with this methodology, respondents were asked to consider burden of illness, inequalities, cost to NHS and impact on family and child when submitting topics for research and public awareness.

In addition, a comment box was provided for HCPs to share any issues, which they have encountered during discussions or consultations between SA children/young people and those caring for them. The survey attached as Supplementary File 1.

#### 2.2.1 Recruitment

All HCPs involved in the care of children were approached to participate in the scoping survey using various different methods. The methods used are listed below:

1. HCPs approached and consented at two different national paediatric conferences
2. Electronic dissemination of the survey link emailed to HCPs by the London Deanery

Through these recruitment processes, approximately 100 HCPs were targeted. Of these, 27 people responded to the scoping survey. This estimates the response rate at 27%.

#### 2.2.2 Analysis

Responses were assembled and categorized to ensure clarity for discussion in the focus groups. Topics were ranked by submission frequency as a precursor to be used as a topic guide in the workshop.

### 2.3 Focus groups of SA children and families

#### 2.3.1 Priorities

The priorities identified in the scoping survey of HCPs were sorted by submission frequency and subsequently compiled into a topic guide for presentation and general discussion in the focus groups. This approach was utilized owing to the anticipated difficulties in a face‐to‐face prioritization exercise between HCPs and SAs.

#### 2.3.2 Setting and participants

To obtain a broader sample of individuals, a wide range of SA individuals from different backgrounds (eg country of origin, reason of migration, religion, ethnicity, disability) and age groups (eg adolescents and elderly) to ensure inclusion of otherwise marginalized SAs. We proactively used members of the SA community to recruit a diverse group of SAs. Co‐ordinators of local centres were enlisted to recruit directly via their networks and to distribute study information to increase awareness of the study. For example, a Pakistani Christian lady who ran youth activities at her local church was enlisted who approached all young families connected with that church to help our study recruitment.

Whilst there were no formal exclusion criteria, a selective approach to recruitment of parents who had children under the age of 10 years or those who self‐reported having children with health issues was made. Parents and guardians who enrolled were invited to bring their adolescent children.

We aimed to be flexible due to the varied availability of participants. The timing of focus groups varied between weekends, evenings and coffee mornings to meet the needs of the participants. A total of 70 participants were recruited of which 35 attended to participate in the focus groups.

Four focus groups were set up, each with seven to 10 participants. Both inner‐ and outer‐city venues that included religious institutions and community centres were utilized to account for SA community dispersion across Leicestershire, UK.

#### 2.3.3 Running of the focus groups

An experienced community facilitator fluent in multiple languages led each focus group. The topics submitted by the HCPs in the scoping survey were then presented to the SAs focus groups, and SAs were asked to prioritize these topics in order of importance to them. The focus groups were also asked to submit health topics important to them, which were not mentioned by HCPs. The community facilitator was provided training on how to separate submissions into research, public awareness and outcome indicator categories.

An observer was present throughout all focus groups to make notes, including notes on group dynamics, and also to help with additional needs of the group. A British Sign Language (BSL) interpreter assisted in the group of hard of hearing SAs, which is novel as there is no published evidence of this particular group of SAs engaging in co‐production studies. An introduction talk explaining the differences between research, public awareness and outcome indicators was given to the focus group participants prior to starting the exercise.

Packs of props that included leaflets on child safeguarding, early starts (ie Best Beginnings), child safety, immunization, organ donation, mental health, vitamin D and disability were utilized as props to facilitate discussions. Each focus group lasted 60‐90 minutes. Participants’ discussions were summarized and then read out. The participants then discussed which topics they considered to be the most important issues. Where possible, participants were asked to think about the order of priority.

#### 2.3.4 Data collection and analysis

A written questionnaire was utilized to collect general demographic data such as age, gender, first language, religion, ethnicity and the number of years living in the UK. No personal identifiers were collected. Assistance was provided when literacy difficulties arose.^10^

Participants were given the option to withdraw from the study at any point and informed that their views would not be considered if they withdrew. None chose to do so. The interview topic guide was piloted in the first focus group. No alterations were considered necessary to the question guide, which is presented in Table 1.

**Table 1:** Focus group question guide

On this whiteboard we've written topics ranked by healthcare Professionals deemed important for research/public awareness to improve the health of South Asian children
Does X topic worry you?
Prompts: yourself; your family; at work; by others (friends, neighbours, the media, “heard about”) the health service.
What specific improvements would you like to see others make in X topic?
Prompts: Western medianes, alternative/complementary mediane, advice from doctors/nurses, leaflets, labelling etc.
How can we tell that we have made a difference? What changes should we measure? What would be measures of success for achieving these improvements?
Prompts: Life expectancy, quality of life etc.
What other health issues which affect South Asian children health not mentioned in this list which you'd like see more research/improvement in public awareness?

Focus group data were organized for analysis after each session between the community facilitator and researcher manually. A content analysis where the key themes and concepts were identified and categorized alongside discussions within groups was undertaken.^38^

Both common categories across groups and categories that were unique to some groups were identified. Frequency counts of issues and views expressed (by type), both in groups and across groups, were also performed. Finally, findings were compared with the scoping survey where concordant and discordant themes between SAs and HCPs were identified.

### 2.4 Ethical approval

Ethical approval for the study was deemed not required by the National Research Ethics Committee (Ref: 04/57) for the purposes of service evaluation. All participants gave informed written consent to participate with all completed consent forms held in a locked cupboard in the research office premises.

## 3 RESULTS

### 3.1 Scoping survey

A total of 27 professionals across the UK responded. These included doctors, nurses, health visitors, psychologists, dentists and social workers. Ranked by submission frequency, topics identified as requiring (i) improved public awareness, (ii) further research and (iii) relevant outcome indicators are listed in Table 2.

**Table 2:** Scoping Survey topics and outcome indicators identified by HCPs

Public awareness	Obesity and diet Mental health illness recognition Health‐care access and health‐seeking behaviour Vitamin D and rickets Routine health checks Allergy and asthma Dental health Diabetes Link between genetic disorders and consanguinity Domestic violence and safeguarding
Research	Nutrition, obesity and physical activity Diabetes Health‐care access and health‐seeking behaviour Health education Parent‐child relationships and child care dynamics Asthma Dental health Infectious diseases
Indicators	Growth, development and physical activity levels Health knowledge School attendance and literacy levels Health‐care utilization Quality of life (QOL) scores Genetic disease rates Diabetes screening participation Morbidity/mortality rates Mental health service uptake Health outcomes

### 3.2 Focus groups

A total of 35 individuals across four focus groups participated. Their demographic details are summarized in Table 3.

**Table 3:** Demographics of the focus group participants

Variable	Focus group 1	Focus group 2	Focus group 3	Focus group 4
Male (n)	2	7	7	2
Age range	18‐46	40‐74	29‐62	16‐57
UK stay length	5‐13	7‐45+	4‐32	16‐57
Setting	Mixed Inner and Outer	Inner city	Inner city	Outer city
Ethnicity	Indian/Pakistani	Indian	Indian	Indian
Religion	Christian	Hindu/MuslimSikh	Sikh	Hindu/Jain
Language	Punjabi/Urdu	BSL	Punjabi	Gujarati
Disability	None	Hard of hard of hearing	None	None

Across all four focus groups, interest was highest on public awareness topics and lowest on outcome indicators. Despite distinct separation of discussions on research and public awareness priorities, participants from all four focus groups chose to merge the discussions citing a striking overlap between both. Lack of awareness of research undertaken by funders such as NIHR, Wellcome Trust and Medical Research Council (MRC) in comparison with Cancer Research UK was cited.

A summary of topics and outcome indicators prioritized by SAs is presented in Table 4. For readability, findings from the focus groups are presented as follows: (i) similarities across focus groups, (ii) differences across focus groups and (iii) differences with scoping survey respondents.

**Table 4:** Topics and outcome indicators prioritized/not prioritized by South Asians

Priorities	Not priorities
1. Concordance and shared decision making	1. Genetic disorders and consanguinity
2. Primary care access	2. Diabetes
3. Mental health	3. Education/Literacy/School attendance
4. Obesity and diet	4. Parenting methods
5. Blood and Organ donation	5. QOL scores
6. Alternative medicine effectiveness	
7. Routine health monitoring	

### 3.3 Similarities across focus groups

#### 3.3.1 Research and public awareness priorities

There were several similarities in priorities between focus groups. All focus groups prioritized obesity and diet as topics requiring further public awareness and research. The importance of intervention at an early age was cited at least once in each group. In particular, focus groups 1 and 2 (Asian Christians and Hard of Hearing Asians, respectively) prioritized increasing awareness surrounding the risks of eating fast food on a regular basis.

Mental health illness recognition was another topic prioritized by all focus groups. Poor awareness surrounding clinical presentation and aetiology of depression as well as misconceptions about people who have psychotic episodes such as “being possessed” appeared to hinder seeking help early, according to focus group 1. Increasing awareness into the link between alcohol and mental health illness was prioritized by focus group 3 (consisting of mixed SAs) and focus group 4 (consisting of Asian Gujaratis)

Access to health‐care and health‐seeking behaviour was prioritized by all focus groups. Interestingly, language barriers and reported racial profiling in emergency departments, and GP practices was felt to cause delay in consenting and receiving treatment according to focus group 1. On the other hand, focus group 2, in particular, mentioned lack of BSL interpreters at appointments leading to delays in treatment. In contrast, increased awareness into taking up routine health screening was more important to focus groups 3 and 4.

Awareness about blood and organ donation was a popular topic prioritized by focus groups 2, 3 and 4. It was felt that advice from religious leaders about organ donation was varied and unclear. According to participants, reasons for not participating in organ and blood donation needed to be explored.

### 3.4 Outcome indicators

As a reflection of the lack of awareness by SAs into health research carried out, participation in discussion about outcome indicators was poor across all focus groups. Focus group 1 noted that school literacy, school attendance and life expectancy were suggested as useful outcome indicators that were easily interpretable. Interestingly, focus group 4, the same group that prioritized increasing awareness into routine health screening, suggested the use of GP referrals to secondary care as an outcome indicator.

### 3.5 Differences across focus groups

Despite the strong similarities across groups, tangible differences were noted in between groups. For example, only focus groups 1 and 4 deemed research and awareness on the effectiveness of alternative medicine as a priority area. In contrast, focus group 3 felt that awareness and research on migrant health should be given key consideration.

It is arguable that the constituents of group of participants can clearly explain marked differences in priorities; for example, research into hearing problems was only prioritized by the focus group consisting of hard of hearing SAs.

Additionally, we noted that inner‐ and outer‐city participants (focus group 3 vs 4) had markedly different views. This depended on how long they had been living in the country, their backgrounds and how they perceived health care. Whilst outer‐city participants consisting mainly of SA Christians thought very highly of HCPs and were equally as keen to engage in joint decision making with them, inner‐city participants prioritized increasing awareness into the availability of health‐care services and less of an emphasis on joint health decision making.

South Asian Christians in the UK are a small group, largely educated and literate. Certain views of focus group 1 consisting of SA Christians differed in relation to the other subgroups. In particular, awareness about blood and organ donation was not prioritized by this group. SA Christians also engaged the most in the focus groups, evidenced by suggestion of research outcome indicators. They also prioritized joint decision making with HCPs as opposed to increasing awareness into the availability of health‐care services compared to the other focus groups.

### 3.6 Differences between SAs and HCPs

There were pronounced differences between topics prioritized by SAs and HCPs. For example, diabetes, vitamin D, rickets and the effect of consanguinity on genetic disease were prioritized by HCPs but not by SAs. In contrast, awareness into the effectiveness of alternative medicines and different parenting methods was prioritized by SAs but not by HCPs. These differences in priorities may represent the importance of involving people from communities whose views are not traditionally considered.

## 4 DISCUSSION

### 4.1 Principal findings

#### 4.1.1 Literature review

There is a considerable amount of literature on prioritization of research on specific disorders or specialist services. It was envisaged that the process of selecting and prioritizing topics that would be included in the focus group and discussions would be influenced by the literature review.

However, it was difficult to relate much of the disease‐specific literature around priority setting to the general health care of SA children and their families. Furthermore, there was little evidence that these research agendas had incorporated the needs of children, adolescents and their families.

In non‐ethnic minority children, preferences towards hospital care (eg food taste, good facility ambience) and doctor‐patient relationship (eg treating them as a responsible adult) were identified.^14^ In contrast, in SA adults, issues regarding immigration and acculturation were noted.^39^ Although important, these were issues that are not easily prioritizable.

As consequence, in keeping with other prioritization exercises, a scoping survey was undertaken prior to focus groups to generate a list of prioritizable topics and outcome indicators.

### 4.2 Prioritization exercise

#### 4.2.1 Mental health illness recognition

Research carried out by the Time to Change partner, Rethink Mental Illness, which looked at attitudes towards mental illness in the SA community noted that mental illness remains a markedly taboo subject.^29^ This bore strong similarities with what was raised by our focus group participants that included shame surrounding mental health illness, causes of mental health illness being misunderstood, families being either extremely caring or isolating, loss of value and damaged marriage prospects.

In a recent review on the research on mental health in SA women in the UK, higher prevalence of depression, suicide and deliberate self‐harm in the SA community was identified.^40^ In concordance with the issues raised by our focus group participants, there is therefore a strong impetus to increase awareness about culturally sensitive mental health services for UK SAs.

### 4.3 Obesity, diet and diabetes

Evidence has consistently noted that SA children are more obese and have a higher rate of diabetes than their White counterparts.^37^, ^41^ The tendency to insulin resistance observed in British SA adults appears to be more apparent in children where an increased sensitivity to adiposity is hypothesized.^42^

Action to prevent non‐insulin‐dependent diabetes in SA adults therefore needs to begin during childhood. Whilst obesity and diet were concordant themes between HCPs and SAs, interestingly diabetes was not. This may be explained by the lack of awareness amongst SAs about the relationship between insulin resistance, adiposity, poor nutrition and their long‐term health consequences.

### 4.4 Organ and blood donation

A campaign launched by the NHS specifically targeting black and ethnic minorities may have contributed to obstacles to organ donation being prioritized by both groups. Research shows that religion is often a barrier to people agreeing to organ donation because they feel their faith does not allow it.^27^ Whilst tackled to a certain extent by the NHS Blood Transfusion campaign, increased awareness on religious viewpoints on organ donation is required.^28^, ^39^

### 4.5 Alternative medicine

Awareness into the use and effectiveness of alternative medicine may have been prioritized by our focus group participants due to the large influence of such medicine in the lives of SAs. There is evidence that older SA family members consider alternative medicine as viable treatment options for chronic conditions such as epilepsy.^26^ Whilst alternative medicine was deemed important to SAs, they were not prioritized by our HCP participants who felt it was not their “business to discuss this.” HCPs feel they lack sufficient training or knowledge on the use of herbal medicines;^43^ perhaps this may affect why increasing the awareness of alternative medicine does not come up as a priority for HCPs. Given the chance, it appears that SAs would be keen to discuss such therapies with their HCPs.

### 4.6 Lack of awareness of current research

There was reduced vocalization by SAs on research compared to public awareness priorities. There is therefore a need to improve public awareness into research carried out by all research funders such as the NIHR, MRC and Wellcome Trust. Consideration should also be given when disseminating research findings to SAs to aid decisions surrounding SA health. Finally, more research is required on the most appropriate methods to inform SAs on the importance of both health research and the use of outcome indicators that matter to SA children and their families.

### 4.7 Implications for clinicians and policy‐makers

This is the first study that has aimed to synthesize the literature and engage SA children, adolescents, families and HCPs in setting priorities for research and public awareness in the health of SA children. We have developed a method that can now be utilized by others who wish to work with different marginalized communities.

By involving both HCPs and multicultural focus groups, we have identified both the mutual concerns and also the divergence of views that exist between SA communities as well as between lay people and HCPs. Although we identified significant commonality in the priorities of the different cultural groups, we also identified differences between them that may have been influenced by ethnicity, culture and disability.

Whilst Research Advisory Committees of major funders such as NIHR, Wellcome Trust and MRC make reference to the need for interventions to be tailored for the particular circumstances of certain ethnic groups, little has been performed in identifying research priorities pertinent to specific cultural groups.

The discordant views between HCPs and SA individuals together with poor awareness of existing UK research funding raise an important dilemma. If scarce resources are to be funnelled towards addressing an expert‐led agenda with its predominantly scientific priorities, then public engagement is likely to be low.

On the other hand, whilst investment in a programme of patient‐identified research and public awareness priorities is more likely to generate satisfaction and engagement, it is not clear how many user perspectives should be taken into consideration to accurately prioritize the needs of UK SAs, a culturally diverse population. Further exploration and development of methods to engage this hard to reach group may be worthwhile.

### 4.8 Refining the co‐production exercise for the future

We have realized that undertaking a project of this nature is both time consuming and difficult to carry out with numerous methodological challenges resulting in several study limitations. In the future, it may be worth considering incentivising responses by HCPs to the scoping survey, to increase the response rate.

In keeping with the existing literature, focus group participants found difficulty in distinguishing research from service delivery and public awareness priorities. Additionally, several other issues such as lack of awareness of current research priorities and difficulty in translation of issues of daily lives into well‐structured research and public awareness priorities were noted. This made formal ranking of priorities difficult. This needs to be considered when undertaking prioritization exercises beyond a pre‐specified disease area asthma for example.

Given the paucity of published literature on the research and public awareness priorities of SAs, it was necessary to undertake an exploratory study that both allowed SAs to raise their own issues but limited in sample size to manage methodological challenges. Even though “grey literature” was searched for from different organizations, extending the literature search to hand searching for specialist journals and reports may have highlighted local projects on SA health priorities. The search terms may also be extended to including terms such as “ethnicity, language, Muslim/Islam, Punjabi, Urdu, Gujarati” which would be pertinent if repeating a similar study.

By selectively recruiting SAs across all ethnicities, religion and disability irrespective of language spoken, we aimed to ensure that the products of our work would be applicable to a culturally diverse population that frequently included under‐researched communities.^4^, ^29^, ^44^

However, sampling bias is certain to ensue, as typically highly motivated individuals, unrepresentative of the general population engage in health‐care decision making. A recurring barrier to participation noted more frequently in the inner city was the perception of “why should I participate if my child is now well” or “by the time any change happens our children would've become adults.” Some of this sampling bias was overcome by the use of a multilingual community facilitator encouraging engagement from the local communities, as opposed to academics inviting the general population to participate.

A limitation of this study is that the educational status of the participants during the focus groups was not collected. In numerous studies on ethnic minorities’ understandings of health and health‐care issues, it emerges that the participants’ educational background may affect these understandings; for example, in the study by Li et al.,^45^ it emerged that the educational background of elderly Chinese migrants living in the UK may have affected their understanding of Western notions of mental illness. This may also be an explanation as to why SA Christians had differing views from other focus groups. In the future, dat on educational status during focus groups should be obtained in a sensitive manner so as not to discourage participation.

Problems can arise when researchers are not fluent in the language or knowledgeable about the culture of the groups that they are involved with. This may lead to an inhibited discussion with these communities.^3^ Additionally, the perceived identity of the researcher or facilitator may further inhibit access to and/or participant recruitment.^46^ Although we aimed to minimize this through the use of a multilingual local community facilitator, this is unlikely to be removed and therefore needs to be considered in the interpretation of findings from this study. Having said that, after the initial hesitation, the majority of respondents were keen to be contacted again in the future for further studies.

In the future, an introductory talk to the community including examples of research, public awareness and examples of projects with outcomes may improve participation. Community mobilization events and engaging with community leaders in the future may also result in increased participation in this type of project.

## 5 CONCLUSION

Decisions around service and research investment towards ethnic minorities by funding bodies have, to date, largely been determined through topic generation from within the biomedical community and health service providers. This has therefore led to a relative imbalance in monies allocated to addressing the priorities of SA children, their families and minority communities.

Our study illustrates that, contrary to common perceptions, SA adolescents and families can constructively engage in priority setting in health and health care. Whilst methodological challenges remain, efforts to engage this diverse but traditionally marginalized group should be emphasized to ensure resource deployment to areas that matter to SA children and their families as well as researchers and health service providers.

## AUTHOR CONTRIBUTIONS

LM, GS and ML conceived and participated in the design of the study with RS, KR and GS coordinating the study. LM and RS wrote the manuscript with all authors helping to draft, read and approve the final manuscript.

## COMPETING INTERESTS

All authors have completed the ICMJE uniform disclosure at www.icmje.org/coi_disclosure.pdf and declare funding of direct research costs for the submitted work from the SAHF. LM and ML (Chair) are unpaid members of the SAHF Child Health Working Group.
